# Polydopamine-Coated Copper-Doped Mesoporous Silica/Gelatin–Waterborne Polyurethane Composite: A Multifunctional GBR Membrane Bone Defect Repair

**DOI:** 10.3390/jfb16040122

**Published:** 2025-04-01

**Authors:** Mengmeng Jin, Yi Hou, Feiwu Kang

**Affiliations:** 1 Department of Oral and Maxillofacial Surgery, Stomatological Hospital and Dental School, Tongji University, Shanghai 200072, China; jinmm2096@163.com; 2 Shanghai Engineering Research Center of Tooth Restoration and Regeneration, Shanghai 200072, China; 3 State Key Laboratory of Oral Diseases, National Clinical Research Center for Oral Disease, Department of Oral and Maxillofacial Surgery, West China Hospital of Stomatology, Sichuan University, Chengdu 610041, China

**Keywords:** guided bone regeneration (GBR), GBR membrane, bone repair, composite biomaterial

## Abstract

Guided bone regeneration (GBR) membrane has proven to be a fundamental tool in the realm of bone defect repair. In this study, we develop a mussel-inspired composite biomaterial through polydopamine-assisted, combining gelatin–WPU matrix with the ion-release behavior of Cu–MSNs for augmented bone regeneration. The optimized composite membrane exhibits enhanced mechanical stability, demonstrating a tensile strength of 11.23 MPa (representing a 2.3-fold increase compared to Bio-Gide^®^), coupled with significantly slower degradation kinetics that retained 73.3% structural integrity after 35-day immersion in physiological solution. Copper ions act as angiogenic agents to promote blood vessel growth and as antimicrobial agents to prevent potential infections. The combined effect of these components creates a biomimetic environment that is ideal for cell adhesion, growth, and differentiation. This research significantly contributes to the development of advanced biomaterials that combine regeneration and infection-prevention functions. It provides a versatile and effective solution for treating bone injuries and defects, offering new hope for patients in need.

## 1. Introduction

Alveolar bone resorption or loss, caused by trauma, tumors, and inflammatory diseases, can have detrimental effects on aesthetic appearance, physiological functions, or tissue repair [1,2,3]. While autografts and allografts are considered as the “gold standard” for repairing alveolar bone defects, their application is limited by donor shortage and site-related morbidity [4]. To overcome these limitations, the guided bone regeneration (GBR) technique has been devised and widely adopted in periodontal, alveolar, and implant surgical procedures [5,6] in which the GBR membrane acts as a physical barrier to exclude soft tissue invasion while promoting osseous defect reconstruction [7,8]. As reported, the global market of such membranes for dental implantation has achieved a substantial profit of about $80 billion USD in 2022 [9]. Among available options, collagen-based membranes, like Bio-Gide^®^, have found extensive clinical application due to their soft tissue exclusion capability and bone-regeneration enhancement [10].

However, their poor mechanical stability and rapid biodegradation within 30 days hinder their effectiveness in alveolar bone repair, and collagen membranes lack inherent antibacterial functionality [11,12,13]. In contrast, synthetic polymers like water polyurethane (WPU), polylactic-*co*-glycolic acid (PLGA), and polycaprolactone (PCL) exhibit high mechanical properties and reduced degradation rates, making them promising candidates for GBR membrane composites [14,15,16]. Nevertheless, excessively high mechanical strength may lead to soft tissue dehiscence, disrupt the healing process, and elevate the likelihood of infection. Hybrid membranes, combining natural and synthetic polymers, have been shown to balance degradation rate and mechanical strength, making them suitable for bone-regeneration environments [17,18,19].

Building on biomaterial innovations, gelatin, a denatured collagen derivative, retains collagen’s favorable characteristics of biocompatibility and bioactivity while eradicating its antigenicity [20]. But its limited mechanical strength restricts its application in load-bearing situations. To address this gap, our laboratory pioneered the modification of gelatin with WPU, creating composite scaffolds that synergize biological and mechanical advantages [21]. Further enhancement was achieved through polydopamine (PDA) coating, leveraging its unique adhesiveness, biocompatibility, and drug-loading capacity for surface functionalization [22,23].

Despite material advancements, infection is a major cause of GBR failure, particularly in the bacterial environment of the mouth [8]. In addition, osteogenesis and angiogenesis are crucial factors in the regeneration process of alveolar bone [24]. While antibiotics like metronidazole, doxycycline, and levofloxacin have been incorporated into biomaterials to exert antibacterial effects, bacteria can develop resistance to these drugs. Emerging solutions employ bioactive ions (Cu^2+^, Ag^+^, Zn^2+^) that combine antibacterial action with tissue-regenerative effects. Notably, Cu^2+^ demonstrates multifunctional capacities, including angiogenesis promotion and immunomodulation. Research has demonstrated that Si^4+^ can also increase the expression of HIF-1α and VEGF [25]. However, uncontrolled release of copper ions risks cytotoxicity, necessitating advanced delivery systems like copper-doped mesoporous silica nanospheres (Cu–MSNs) for sustained ion release [26].

On the whole, contemporary GBR membranes face three principal limitations: (1) the trade-off between strength and degradation rate, (2) inadequate bioactivity for simultaneous anti-infection and tissue regeneration, and (3) poor adaptivity to dynamic healing microenvironments. To address these, next-generation designs employ (i) Janus-structured membranes with spatially graded mechanical properties, (ii) bioactive ion-releasing nanocomposites, and (iii) stimulus-responsive materials adapting to physiological changes.

**Figure 1:**
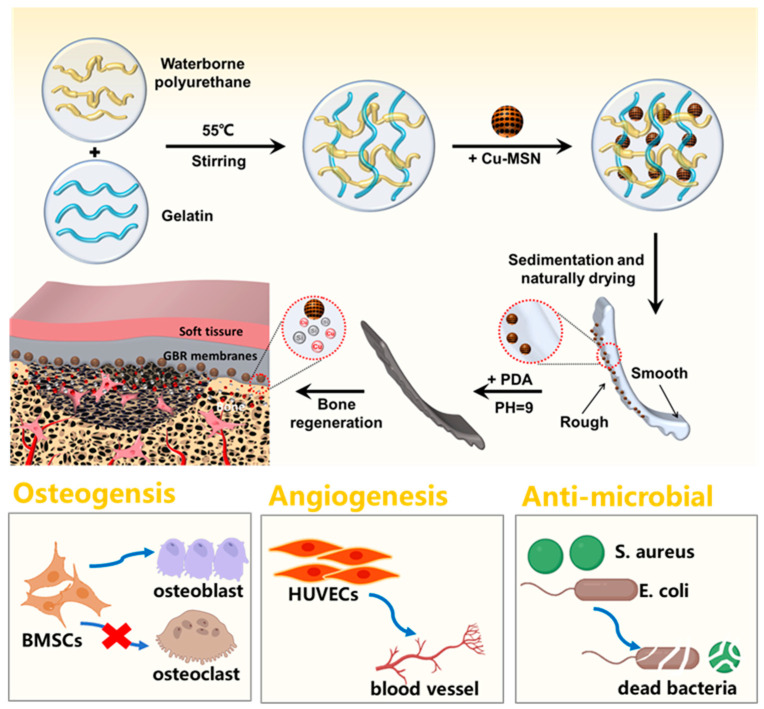
Schematic representation of Cu–MSN/GP–PDA GBR membrane and its biological effects.

Our approach integrates these strategies through rational material hybridization and surface engineering. In this study, we constructed an asymmetric membrane using the solvent casting method (Figure 1). The matrix material was composed of Cu–MSN, gelatin, WPU composite, and PDA coating, which has good biocompatibility and mechanical properties. The design incorporates three key innovations: (1) a Cu–MSN reinforced matrix providing sustained Cu^2+^/Si^4+^ release for angiogenic-osteogenic-antibacterial tri-functionality, (2) PDA surface coating enhancing hydrophilicity, cell adhesion, and antioxidant capacity, and (3) spontaneous nanoparticle settling creating asymmetric surface to guide differential cellular responses. This multifunctional membrane aims to overcome existing limitations while maintaining essential mechanical integrity for alveolar bone regeneration.

## 2. Materials and Methods

Copper nitrate trihydrate (Cu(NO_3_)_2_·3H_2_O), cetyltrimethylammonium bromide (CTAB), tetraethyl orthosilicate (TEOS), ammonium hydroxide(NH_3_·H_2_O), isophorone diisocyanate (IPDI), N,N-dimethylformamide (DMF), triethylamine (TEA), gelatin, dopamine hydrochloride, and Tris-HCl buffer were sourced from Shanghai Aladdin Co., Ltd., (Shanghai, China). 2,2-dimethylol propionic acid (DMPA), poly(tetramethylene ether glycol) (PTMG, Mw 2000), and pentobarbital sodium were purchased from Sigma-Aldrich (St. Louis, MO, USA). Double-distilled water was homemade in the lab, and ethanol was sourced from Sinopharm Chemical Reagent (Shanghai, China). Phosphate buffer solution (PBS), alpha Modification Eagle Medium (α-MEM), penicillin-streptomycin, and Dulbecco’s Modified Eagle’s Medium (DMEM) were purchased from HyClone (Logan, UT, USA). Fetal bovine serum was purchased from Zhejiang Tianhang biotechnology Co., Ltd., (Hangzhou, China). 4% paraformaldehyde, ethylene diamine tetra-acetic acid (EDTA), and Hematoxylin-Eosin stain kit were procured from Beijing Solarbio Science & Technology Co., Ltd., (Beijing, China). The live/dead assay kit was obtained from APExBIO (Houston, TX, USA), cell counting kit 8 (CCK-8) was obtained from KeyGEN BioTECH (Nanjing, China), and crystal violet was obtained from Beyotime (Shanghai, China). LB Agar Powder and LB sterile liquid medium were obtained from Sangon Biotech Co., Ltd., (Shanghai, China).

### 2.1. Synthesis of Cu–MSNs

For the synthesis of Cu–MSNs, begin by dissolving 1.8 g of CTAB in 500 mL of distilled water, adding 10 mL ammonium hydroxide drop by drop and stirring at 80 °C for 1 h. Dissolve 0.5 g of Cu(NO_3_)_2_·3H_2_O in 1 mL of ethanol to create a clear, dark blue solution, which is then mixed with 9 mL of TEOS under stirring and ultrasonic treatment for 15 min [27]. Gradually add this homogeneous solution to the CTAB preparation while stirring vigorously. Continue stirring for an additional 4 h, then collect the synthesized nanospheres via centrifugation at 8000 rpm for 20 min. Wash the nanospheres three times with distilled water and ethanol, respectively. Freeze-dry the precipitate of Cu–MSNs for 12 h and calcine them in air at 600 °C for 6 h to eliminate any remaining CTAB [26].

### 2.2. Synthesis of WPU Dispersions

12.00 g PTMG-2000 and 6.67 g IPDI were mixed for stirring 2.5 h, at 85 °C under nitrogen atmosphere. Subsequently, 1.2 g of DMPA, dissolved in 5 mL of DMF, was added and stirred at 62 °C for 1 h. Following this, 1.2 g of TEA was incorporated into the mixture and stirred for an additional 15 min. After adding distilled water and stirring at room temperature for 1 h, a 30 wt% PU water emulsion was obtained [28].

### 2.3. Preparation of Gelatin–WPU Membranes and Cu–MSN Doped Gelatin–WPU Membranes

Weigh 10 g WPU emulsion (30% solid content), mix it by ultrasound, and stir at room temperature. Mix 0.6 g gelatin and 3 mL distilled water into the WPU emulsion (gelatin: WPU = 1:5), and heat and stir at 55 °C until the gelatin is completely melted. Afterwards, pour the mixed emulsion into a glass dish. The dispersions were allowed to dry at room temperature for 24 h, followed by an additional 4 h of drying in a vacuum oven at 85 °C until the gelatin–WPU (GP) membranes achieved a constant weight. The dried GP membranes, approximately 1 mm thick, were then stored for future use. According to the ratio of solid content of gelatin and WPU, the different composite membranes were named as GP1 (1:1), GP3 (1:3), and GP5 (1:5).

The process of Cu–MSN doped GP membrane is similar to GP membranes. Cu–MSN was dissolved in distilled water and mixed evenly by ultrasound. Add Cu–MSN solution drop by drop to WPU emulsion, stir well, then add gelatin, stir at 55 °C until the mixture is uniform. The dispersions were first dried at room temperature for 24 h, followed by an additional 4 h in a vacuum drying oven at 85 °C, until the Cu–MSN/GP membranes attained a stable weight. The dried Cu–MSN/GP membranes were then kept for future applications.

### 2.4. Preparation of PDA Coated Cu–MSN/GP

A solution of dopamine hydrochloride was prepared at a constant concentration of 2 mg/mL in a basic (pH = 9) Tris-HCl buffer (10 mM). The membranes were then submerged in this solution. The self-polymerization of dopamine, triggered by the pH of the solution, caused it to turn dark brown. The membranes were allowed to react in the medium at room temperature for 8 h. Afterward, the coated membranes (Cu–MSN/GP–PDA) were rinsed with double-distilled water at least three times, dialyzed for 3 days, and finally freeze-dried.

### 2.5. Material Characterization

#### 2.5.1. Characterization of Cu–MSN/GP–PDA Membranes

The surface morphologies of the GP, Cu–MSN/GP, and Cu–MSN/GP–PDA membranes were examined using a scanning electron microscope (SEM, JSM-6500LV, Tokyo, Japan) at an accelerating voltage of 3 kV, after the samples were coated with gold (Au). To analyze the elemental composition (C, O, N, Si, and Cu) of Cu–MSN, an energy-dispersive X-ray spectrometer (EDS, Elite T, Waltham, MA, USA) attached to the SEM was utilized. Furthermore, a transmission electron microscope (TEM, JEM-2100Plus, Tokyo, Japan) was employed to delve deeper into the microstructure of Cu–MSN. Three non-overlapping TEM micrographs were acquired at 100,000× magnification, followed by scale calibration. In Image J (V1.8.0.112), 100 isolated particles were manually measured to calculate diameters. Export the data through excel and calculate the mean value and standard deviation, with results expressed as mean ± SD. The composition of the Cu–MSN/GP–PDA membrane was assessed through Fourier transform infrared spectroscopy (FTIR, INVENIO-R, Ettlingen, Germany). The FTIR spectra of various samples were recorded using the potassium bromide (KBr, supplied by Aladdin, West Palm Beach, FL, USA) pellet method, within a wavenumber range of 4000 to 400 cm^−1^. Additionally, an X-ray diffractometer (XRD, Empyrean, Almelo, The Netherlands) was used to investigate the phase composition and crystallinity of the Cu–MSN/GP–PDA membrane, with a scanning range of 10 to 60 degrees [29].

#### 2.5.2. Mechanical Property

The tensile properties of the Cu–MSN/GP–PDA GBR membrane were evaluated using an electronic universal material testing machine (Instron 5967, Norwood, MA, USA), equipped with a 250 N sensor and operating at a speed of 10 mm/min [29]. Each sample was cut into rectangular strips measuring 15 mm × 4 mm (length by width) and securely mounted vertically on the clamping device of the tester. The Young’s modulus, elongation at break, break energy, and tensile strength of the membranes were determined based on the tensile stress-strain curve derived from the instrument’s data.

#### 2.5.3. Hydrophilic Property

The surface hydrophilic property of the fabricated films was evaluated through static contact angle measurements using distilled water droplets. An optical contact angle goniometer (Krüss DSA100, Hamburg, Germany) was employed to quantify the contact angle, defined as the angle formed between the horizontal film surface and the tangent line of the liquid-air interface at the three-phase contact point. To ensure measurement reliability, three independent measurements were performed at different surface locations, and the average value was reported.

#### 2.5.4. In Vitro Degradation and Ions Release Property

Square samples of 10 mm × 10 mm were cut from the various membrane specimens, and their initial weights (m_0_) were recorded. These samples were then placed in centrifuge tubes containing 10 mL of PBS solution (0.1 M, pH 7.4) and incubated at 37 °C with a shaking speed of 90 rpm. Every 3 days, the PBS solution was replaced, and the freeze-dried weights (m_t_) of the samples were measured on days 1, 7, 14, and 35. The weight-loss rate of the membranes was calculated using the formula: Weight loss rate (%) = [(m_0_ − m_t_)/m_0_] × 100%.

Additionally, 10 mm × 10 mm square samples were cut from different membrane specimens and placed in centrifuge tubes filled with 5 mL of distilled water. These tubes were then incubated at 37 °C with a shaking speed of 90 rpm. At specific time points (1, 4, and 7 days), 5 mL of the solution was removed and replaced with an equal volume of fresh distilled water. The concentrations of Si and Cu ions in the collected solutions were measured using inductively coupled plasma mass spectrometry (ICP-MS, 5100 SVDV, Santa Clara, CA, USA).

### 2.6. In Vitro Biocompatibility Assay

Human umbilical vein endothelial cells (HUVECs) were purchased from Univ Biotechnology Co., Ltd., (Shanghai, China) and cultured in DMEM with 10% fetal bovine serum (FBS), 1% penicillin-streptomycin at 37 °C in 5% CO_2_ atmosphere. Rat bone marrow mesenchymal stem cells (rBMSCs) were extracted from the bone marrow of 7-day-old SD rats and cultured in α-MEM with 10% fetal bovine serum (FBS), 1% penicillin-streptomycin at 37 °C in 5% CO_2_ atmosphere.

The prepared Cu–MSN/GP–PDA membrane was evaluated for cytotoxicity using the Cell Counting Kit 8 (CCK-8, APExBIO, USA) method and Live/Dead staining (KeyGEN BioTECH, KGAF001, China). The membrane was soaked in the complete medium for 24 h, and the soaked medium was collected. rBMSCs (1 × 10^4^ cells/mL) and HUVECs (5 × 10^3^ cell/mL) were seeded in 96-well plates (100 μL per well) and incubated in a humidified environment at 37 °C, with 5% CO_2_ concentration. The culture medium was replaced every 48 h. Following 1 and 4 days of incubation, the samples were exposed to CCK-8 solution for 3 h, and the absorbance at 450 nm was determined using a microplate reader (SpectraMax 190, San Jose, CA, USA). Additionally, Live/Dead staining was employed to assess cell viability after 1 and 2 days of culture.

### 2.7. Transwell Assay

For the transwell migration assay, 200 µL of serum-free medium containing 2 × 10^4^ HUVECs was dispensed into the upper chamber of the transwell inserts (Corning). Each lower chamber was equipped with a 5 mm × 5 mm square membrane and 500 µL of DMEM. Following a 24 h incubation period at 37 °C, cells that had not migrated and remained on the upper surface of the 8 µm pore filter membrane were gently removed using a cotton swab. Subsequently, the cells were washed with PBS and fixed in 4% paraformaldehyde for 15 min, after which they were stained with crystal violet for an additional 10 min. The number of cells that had successfully migrated through the filter membrane was counted and documented through photographic images captured using an inverted optical microscope.

### 2.8. Anti-Bacterial Test

The Cu–MSN/GP–PDA membrane’s antibacterial activity was evaluated by Gram-negative *Escherichia coli* (*E. coli*, ATCC 25922) and Gram-positive *Staphylococcus aureus* (*S. aureus*, ATCC 25923) bacteria in vitro. A 5 mm × 5 mm square membrane was placed in each well of a 48-well culture plate and subjected to ultraviolet (UV) irradiation for 1 h. Subsequently, 500 μL of bacterial suspension (1 × 10^7^ CFUs/mL) was added to each well and cultured at 37 °C. After 24 h, 100 μL of bacterial liquid was drawn from each well, the liquid gradient was diluted to 10^−8^, and 10 μL of each dilution was plated onto solid agar plates. Following a 12 h incubation at 37 °C, images of bacterial colony formation were captured to document the effects of the different treatments.

### 2.9. In Vivo Assay

The GBR membranes utilized were initially subjected to UV-sterilization to eliminate any potential contaminants. Nine male rats, weighing between 200 and 220 g, were maintained under standard conditions (*n* = 9). Initially, they were anesthetized through intraperitoneal injection of 1% pentobarbital sodium solution. For implantation purposes, the dorsal skin of the rats was shaved and incised using a sterile scalpel to create a pocket measuring 1 square centimeter. Subsequently, membranes, each measuring 5 mm × 5 mm, were inserted into the pocket, and the incisions were closed using sutures. Each rat received three implants, and after 7 days, three rats from each group were selected for sample retrieval.

The animal experiments adhered to international guidelines on animal welfare and met the standards set by the Animal Research Committee of the State Key Laboratory of Oral Diseases and West China School of Stomatology (Approval NO. WCHSIRB-D-2022-604 and date of approval 10 November 2022). A total of nine female Sprague–Dawley rats, sourced from Dashuo in Chengdu, China, were used, with an average weight ranging from 200 to 220 g. The handling of animals and the surgical procedure have been detailed in previous publications. Briefly, the surgical process was conducted under anesthesia induced by intraperitoneal injection of 1% pentobarbital sodium. A defect was created in each femoral condyle using a trephine with an internal diameter and penetration depth of 3 mm, while the site was generously irrigated with 0.9% NaCl. With the exception of the control group, each defect was covered with either a GP membrane, a Cu–MSN/GP membrane, or a Cu–MSN/GP–PDA membrane. The four experimental groups were randomized to ensure an equal distribution among the animals and between the right and left locations. The retrieval procedure was performed at 4 weeks, and the collected samples were then immersed in formalin (*n* = 3 per group).

### 2.10. Micro-CT Analysis

At 1 month after implantation, the rats’ femurs were harvested for Micro-CT (Quantum GX II, Hopkinton, MA, USA) analysis. Three-dimensional (3D) images of both the overall structure and longitudinal sections were captured. Utilizing the Living Image^®^ Software (V4.5), morphometric data such as bone volume fraction (BV/TV), trabecular number (Tb.N), and trabecular separation (Tb.Sp) were extracted from the region of interest and analyzed. These data were then compared to assess the bony alterations among the various groups.

### 2.11. Histological Examination

The tissue samples obtained were preserved in 4% paraformaldehyde for a duration of 3 days and subsequently underwent decalcification using 15% ethylene diamine tetra-acetic acid (EDTA). The samples were then progressively dehydrated with ethanol and embedded in paraffin. Using a microtome manufactured by Leica, Bergen County, NJ, USA, the embedded samples were sectioned to a thickness of 5 μm and mounted onto slides. Subsequently, the sections were stained with hematoxylin and eosin. Ultimately, these stained sections were observed and analyzed under a Leica microscope.

### 2.12. Statistical Analysis

All measurements were conducted at least three times, and the quantitative data are reported as the mean ± standard deviation. For statistical analysis, one-way analysis of variance (ANOVA) followed by Tukey’s post hoc test was employed using the Graphpad Prism (V9.5.0) software. The levels of statistical significance were set at * *p* < 0.05, ** *p* < 0.01, and *** *p* < 0.001.

## 3. Results and Discussion

### 3.1. The Morphology and Characterization of Cu–MSN and Cu–MSN/GP–PDA Membranes

**Figure 2:**
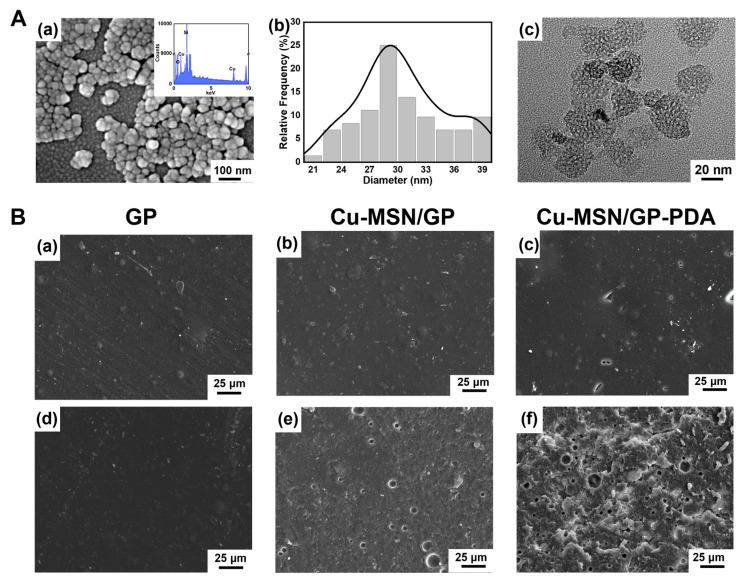
The morphology and composition. (**A**) Cu–MSNs. (**a**) SEM and EDS, (**b**) Particle size distribution, (**c**) TEM. (**B**) SEM of GP (**a**,**d**), Cu–MSN/GP (**b**,**e**), Cu–MSN/GP–PDA (**c**,**f**), the smooth surface (**a**–**c**), and the rough surface (**d**–**f**).

SEM images revealed that all the Cu–MSNs had a uniform spherical morphology (Figure 2A(a)) and the average diameter of the Cu–MSNs is 28.74 ± 4.64 nm (Figure 2A(b)). To verify the incorporation of copper ions into MSNs, samples were investigated by EDS. Cu was detected in the nanospheres, and the weight percentage of Cu is about 6.7%. TEM showed Cu–MSNs had ordered mesoporous structure (Figure 2A(c)), which was due to the CTAB was used as the mesopore template in the preparation phase. The above results indicated the successful preparation of Cu–MSNs.

As SEM shown in Figure 2B, the both sides of GP membrane had smooth surface, while the two sides of Cu–MSN/GP and Cu–MSN/GP–PDA were different. The air-contact surface was smooth, but the substrate-contact surface was rough. It is gravity that caused some Cu–MSNs agglomerate together and settle to the bottom layer [30,31]. The deposition of Cu–MSNs increased the polar groups on the substrate-contact surface, and then increased the surface energy, which was conducive to the deposition of PDA [32]. As shown in Figure 2B(f)**,** there are much tiny flake structures on the substrate-contact surface, which are presumably caused by the PDA layer falling off and adhering to the surface.

**Figure 3:**
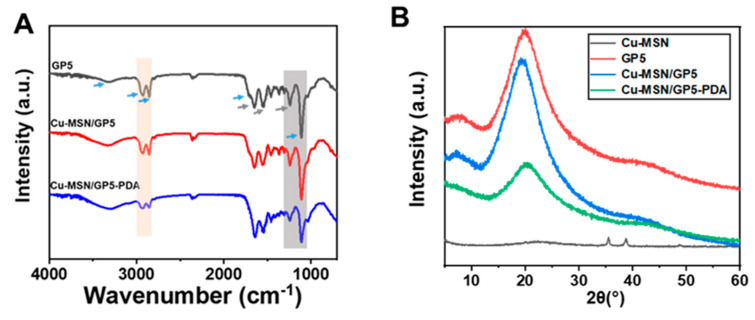
Characterization of Cu–MSN, GP5, Cu–MSN/GP5 and Cu–MSN/GP5–PDA. (**A**) FTIR (blue arrow: key structure of the polyurethane, black arrow: key group of the gelatin), (**B**) XRD.

In order to prove the successful synthesis of the membranes, FTIR was used to characterize its chemical structure (Figure 3A). The typical characteristic peaks of gelatin are reflected in the spectrum. The peaks at 1651 cm^−1^ (amide I), 1551 cm^−1^ (amide II), and 1240 cm^−1^ (amide III), corresponding to C=O stretching, N–H deformation vibration, and -NH bending vibration, respectively [33,34]. The characteristic peaks of polyurethane appear at approximately 3330 cm^−1^ (the tensile vibration of N–H), 2930 cm^−1^, and 2853 cm^−1^ (stretching vibration of C–H), 1718 cm^−1^ (C=O stretching vibration), and 1110 cm^−1^ (C–O–C stretching vibration) [35,36]. In addition, the deformation vibration region of N–H and the stretching vibration region of C–N appeared near 1551 cm^−1^, which proved the formation of a carbamate bond [37]. After the Cu–MSN/GP5 coated with PDA, the peaks at 2930 cm^−1^, 2853 cm^−1^, and 1110 cm^−1^ weakened. The reason may be that dopamine is rich in amino and hydroxyl groups, which can form hydrogen bonds with other molecules [38].

According to the XRD analysis (Figure 3B), the Cu–MSN group had distinct diffraction peak at 2θ = 35°, 38°, and 48°, which indicated CuO can be detected on the samples [39]. The dispersion peaks of gelatin and polyurethane were broad, the peaks of which were 2θ about 20°, indicating that the gelatin and polyurethane were amorphous and had no crystal structure. Cu–MSN/GP5 and Cu–MSN/GP5–PDA also had dispersion peaks, and did not show the characteristic diffraction peak of Cu–MSN, indicating that Cu–MSN interacted with gelatin or polyurethane, and Cu–MSN particles were coated with composite macromolecules.

### 3.2. Mechanical Property

**Figure 4:**
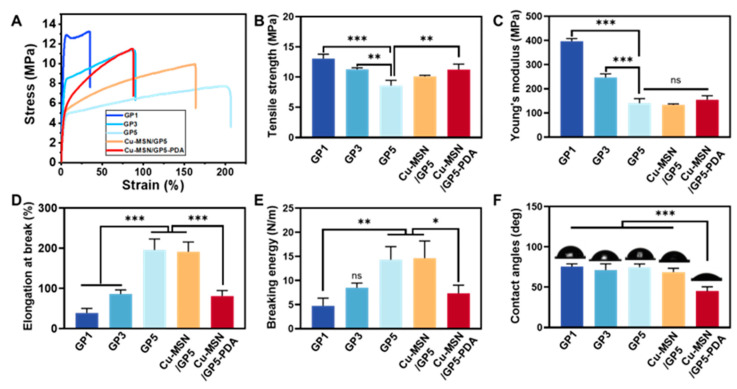
Characterization of mechanical property and Hydrophilic property. (**A**) Representative stress-strain curves, (**B**) ultimate tensile strength, (**C**) Young’s modulus, (**D**) elongation at break and (**E**) breaking energy of the GP1, GP3, GP5, Cu–MSN/GP5, and Cu–MSN/GP5–PDA membranes, (**F**) contact angles of the GP1, GP3, GP5, Cu–MSN/GP5, and Cu–MSN/GP5–PDA membranes. * *p* < 0.05, ** *p* < 0.01, and *** *p* < 0.001. ns means no significant difference.

The excellent mechanical properties of GBR membranes are crucial for maintaining the space beneath them and preventing damage that could compromise their effectiveness. The tensile properties of these different component films are tested by an electronic universal material testing machine. Figure 4A is the typical stress–strain curves of different ratio of gelatin and polyurethane membranes (GP1, GP3, GP5), Cu–MSN/GP5 membranes and Cu–MSN/GP5-PDA membranes. Figure 4B–E corresponds to the ultimate tensile strength, Young’s modulus, elongation at break, and breaking energy, respectively. The soft and hard segments of PU is thermodynamic incompatible, in that PU has the tendency of spontaneous phase separation. The curled molecular chain is stretched along the stress direction in the process of the soft segment chains are pulled out. Meanwhile the hard segment chains are also pulled out. But the intermolecular forces of hard segment chains are larger, and the molecular chain is not easily subject to movement, so the stress gradually becomes larger as the strain increases [40].

The study demonstrated that reduced gelatin ratios resulted in decreased ultimate tensile strength (13.06 ± 0.72 MPa for GP1, 11.29 ± 0.25 MPa for GP3, 8.59 ± 0.86 MPa for GP5) and Young’s modulus (397.07 ± 10.72 MPa for GP1, 247.23 ± 14.52 MPa for GP3, 141.18 ± 18.27 MPa for GP5), whereas elongation at break (38.96% ± 11.23% for GP1, 86.01% ± 10.14% for GP3, 195.56% ± 27.32% for GP5) and fracture energy (4.74 ± 4.51 N/m for GP1, 8.47 ± 4.04 N/m for GP3, 14.32 ± 4.31 N/m for GP5) exhibited significant increases. The addition of gelatin produced a moderate cross-linked structure in the whole system, which effectively reduced the deformation of the composite membranes and improved the strength. Due to the existence of soft segment chains in PU, with the increase of PU content, the elasticity of membranes increases [41]. According to previous literatures, GBR membrane is sufficient to meet clinical requirements when its tensile strength is higher than 3 MPa [42]. When the ratio of gelatin to PU is 1:5, the membrane has appropriate mechanical properties and elasticity, so we chose GP5 in the latter experiments.

Compared with GP5, the tensile stress of Cu–MSN/GP5 (10.11 ± 0.17 MPa) and Cu–MSN/GP5-PDA (11.23 ± 0.91 MPa) is higher. This is mainly due to the dispersion of Cu–MSN nanoparticles in the gelatin–PU matrix, it acts as the crosslinking point and plays an important role in strengthening. The phenolic hydroxyl group of dopamine can chelate the copper ion, which increases the mechanical strength to some extent [43]. PDA coating was prepared in alkaline environment, but WPU will show alkali aging phenomenon in alkaline medium [44]. WPU is diffused and permeated by medium molecules, and its sufficient interaction with molecular chain segments will destroy the secondary valence bonds of macromolecules and break. At the same time, active groups on the PU chain segments, such as ester groups, are more likely to undergo hydrolysis or oxidation reactions, destroying the major bond of macromolecules and reducing the elasticity. So, the elongation at break and breaking energy of Cu–MSN/GP5–PDA (80.98% ± 13.60%, 7.32 ± 1.70 N/m) are lower than GP5 and Cu–MSN/GP5 (191.18% ± 24.26%, 14.63 ± 4.72N/m) (Figure 4D).

Notably, the Cu–MSN/GP5–PDA membrane demonstrated superior mechanical performance compared to the commercial Bio-Gide^®^ collagen membrane, exhibiting enhanced Young’s modulus (154.9 MPa vs. 15.7 MPa), tensile strength (11.23 MPa vs. 3.7 MPa), and elongation at break (80.98% vs. 46.8%) [45]. Two additional commercially available collagen membranes (named Collprotect and Jason) demonstrated Young’s modulus, tensile strength, and elongation at break values of (158.5 MPa, 13.1 MPa, 16.3%) and (178.9 MPa, 13.0 MPa, 17.9%), respectively [46]. Their Young’s modulus and tensile strength were comparable to those of the Cu–MSN/GP5–PDA membrane, while the elongation at break values represented approximately one-fifth of the Cu–MSN/GP5–PDA membrane’s performance. The Cu–MSN/GP5–PDA membrane’s enhanced mechanical profile confers three critical clinical advantages: (1) Surgical stability via tear resistance; (2) Space maintenance through modulus matching; (3) Extended functional durability outperforming resorbable materials’ degradation thresholds [47,48]. This triad ensures stable osteogenic environments with reduced exposure risks.

### 3.3. Hydrophilic Property

The hydrophilicity of membranes has great influence on the adhesion and proliferation of cells. The Cu–MSN/GP5–PDA membranes are hydrophilic with contact angles of about 44.9° ± 5.6°, while other groups (GP1, GP3, GP5, Cu–MSN/GP5) is moderately hydrophilic with contact angle of 75.6° ± 3.4°, 71.1° ± 7.8°, 74.8° ± 4.0°, and 68.8° ± 4.7°, respectively, as shown in Figure 4F. The two sides of all membranes have similar water contact angle. The application of PDA coating notably enhances the hydrophilic properties of the membranes, due to the presence of amine and hydroxyl functional groups within its structure. Furthermore, this heightened hydrophilicity accelerates the biodegradation rate of the membranes and fosters tissue regeneration.

### 3.4. In Vitro Degradation and Ions Release Property

**Figure 5:**
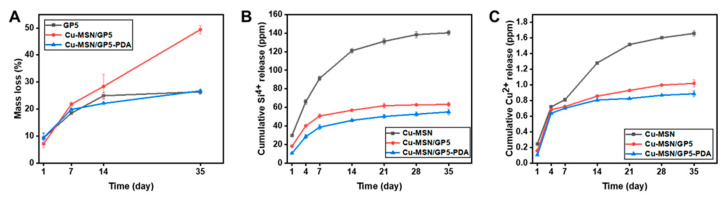
Characterization of degradation and ions release. (**A**) In vitro degradation profiles of the membranes, (**B**) In vitro release behavior of Cu ions, (**C**) In vitro release behavior of Si ions.

The degradation performance of the GBR membrane is a pivotal characteristic. Non-degradable materials necessitate a secondary surgical procedure for removal, and the rate of material degradation significantly influences the process of bone regeneration. The weight-loss rates of different GBR membranes were depicted in Figure 5A. Different from the GP5 membrane, the addition of Cu–MSN resulted in a large increase in the degradation rates of the membranes, from 26.7% to 49.4% at 35d. It is plausible that the incorporation of Cu–MSN disrupts the organized structure of the membrane, accompanied by a relatively rapid degradation rate of Cu–MSN/GP5. The resultant void enhances the interfacial contact between the liquid and the membrane, thereby accelerating the degradation process. Notably, the degradation rates of Cu–MSN/GP5–PDA was similar to GP5, owing to the protective layer provided by the PDA coating. According to the existing literature, complete bone regeneration in critical defects of the femoral condyle is not achievable within 12 weeks. However, in our study, the Cu–MSN/GP5–PDA membrane exhibited a degradation rate of less than 30% after 5 weeks, suggesting its potential for sustained functionality over an extended period. Several studies have shown that the thickness of collagen film decreases significantly, and the degradation rate is too fast at 14–30 days after implantation, while synthetic polymers like PCL degrade excessively slowly in vivo (up to 2–3 years) [49].

As the Figure 5B,C shown, higher concentration of released Cu^2+^ and Si^4+^ were detected in Cu–MSN nanoparticles, and their initial burst release are also more significant. For Cu–MSN/GP5 and Cu–MSN/GP5–PDA membranes, the initial burst release and the release amount of Cu^2+^ and Si^4+^ much lower. By day 35, only 0.89 ppm of the total copper ions are released from Cu–MSN/GP5–PDA membrane, which can achieve slow release and play a long-term role in promoting blood vessels and antibacterial. The release of copper ion is much slower than that of silicon ion, possibly because copper ion can form chelation with polydopamine.

### 3.5. In Vitro Biocompatibility

**Figure 6:**
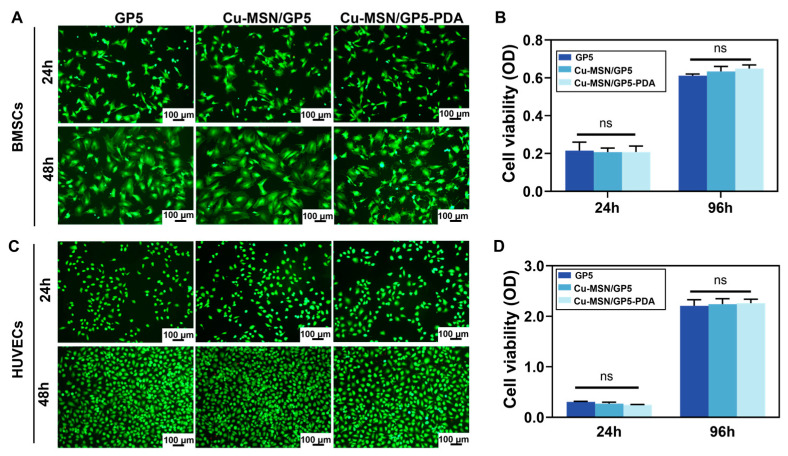
Biocompatibility of the rBMSCs and HUVECs cocultured with the extract of membranes. (**A**) Live/dead staining of rBMSCs, (**B**) CCK-8 analysis of rBMSCs, (**C**) Live/dead staining of HUVECs, (**D**) CCK-8 analysis of HUVECs. ns means no significant difference.

Bone formation involves the recruitment, angiopoiesis, and subsequent osteogenic differentiation of BMSCs, which requires a favorable microenvironment for cell growth. The biocompatibility of membranes is one of the most critical issues to affect its application in bone tissue regeneration. Herein, we chose rBMSCs and HUVECs to investigate the cytotoxicity of various membranes in vitro. As shown in Figure 6, cell viability and proliferative ability of rBMSCs and HUVECs co-cultured with the extract of GP5, Cu–MSN/GP5, and Cu–MSN/GP5–PDA membranes were evaluated by CCK-8 and Live/Dead staining assay. According to the result of Live/Dead staining assay, we can see predominantly live cells (green) and few dead cells (red) on the three groups both BMSCs and HUVECs. At each detection time point, there were no notable differences in OD values among the membranes tested, indicating that the integration of Cu–MSN and PDA coating into the membranes did not adversely affect cell viability. Moreover, a suitable concentration of copper ions does not impede cell proliferation.

### 3.6. HUVECs Migration and in Vivo Angiopoiesis

**Figure 7:**
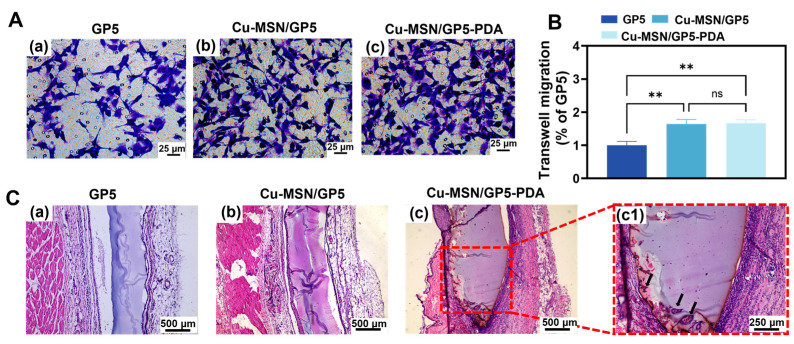
Angiogenic stimulation of the membranes. (**A**) Representative images of transwell assay displaying HUVECs penetrating the filter membrane, under the induction of different membranes, (**B**) For transwell assay, the number of HUVECs passing through the filter membrane was counted, (**C**) In vivo histology changes of subcutaneous tissues after implantation of membranes in a rat model for 7d. ** *p* < 0.01, ns means no significant difference.

As depicted in Figure 7A,B, the Cu–MSN/GP5 and Cu–MSN/GP5–PDA groups exhibited a significant increase in the number of migrated cells during the transwell assay, compared to the GP5 group. Crucially, in a subcutaneous implantation model, the Cu–MSN/GP5–PDA group demonstrated a higher degree of vascularization than both the GP5 and Cu–MSN/GP5 groups, as illustrated in Figure 7C. We can see some cavity structures formed in Cu–MSN/GP5–PDA membrane, while GP5 and Cu–MSN/GP5 did not have similar structures. There are some red cells in the cavity structures, which may indicate the formation of small blood vessel. This may be because the polydopamine coating encourages endothelial cells to adhere to the membrane surface and the release of copper and silicon ions promote blood vessel formation. Collectively, these findings suggest that the Cu–MSN/GP5–PDA composite membrane developed in this study holds promise for inducing neovascularization concurrently.

### 3.7. Anti-Bacterial Test

**Figure 8:**
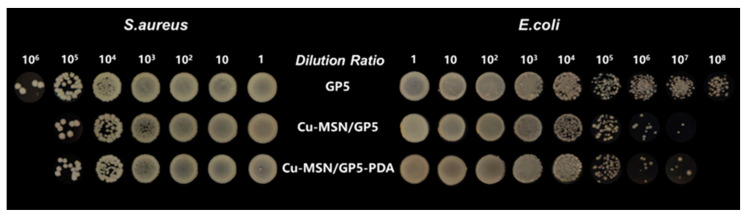
Antibacterial effect of three membranes on *E. coli* and *S. aureus* by gradient dilution method.

The inhibitory effects of the GP5, Cu–MSN/GP5, and Cu–MSN/GP5–PDA groups against both *E. coli* and *S. aureus* were comparatively assessed, with the respective antibacterial efficacy illustrated in Figure 8. After 24 h of co-cultivation, the Cu–MSN/GP5 and Cu–MSN/GP5–PDA groups exhibited remarkable antibacterial activity against both bacterial strains. Conversely, the GP5 group failed to inhibit the proliferation of *E. coli* and *S. aureus*. The results indicated that the release of Cu^2+^ ions exhibits antibacterial properties by either exhibiting toxicity to bacteria or inhibiting their adhesion and proliferation. The key mechanisms involved are: (i) the electrostatic interaction between Cu^2+^ ions and the negatively charged bacterial surface, which disrupts the cell membrane; (ii) the production of reactive oxygen species (ROS) that causes apoptosis by damaging lipids, proteins, membranes, and DNA; and (iii) the disruption of bacterial respiratory chain activity and gene replication processes [50,51].

### 3.8. Regenerative Potential of Membranes in Bone Defect Model

**Figure 9:**
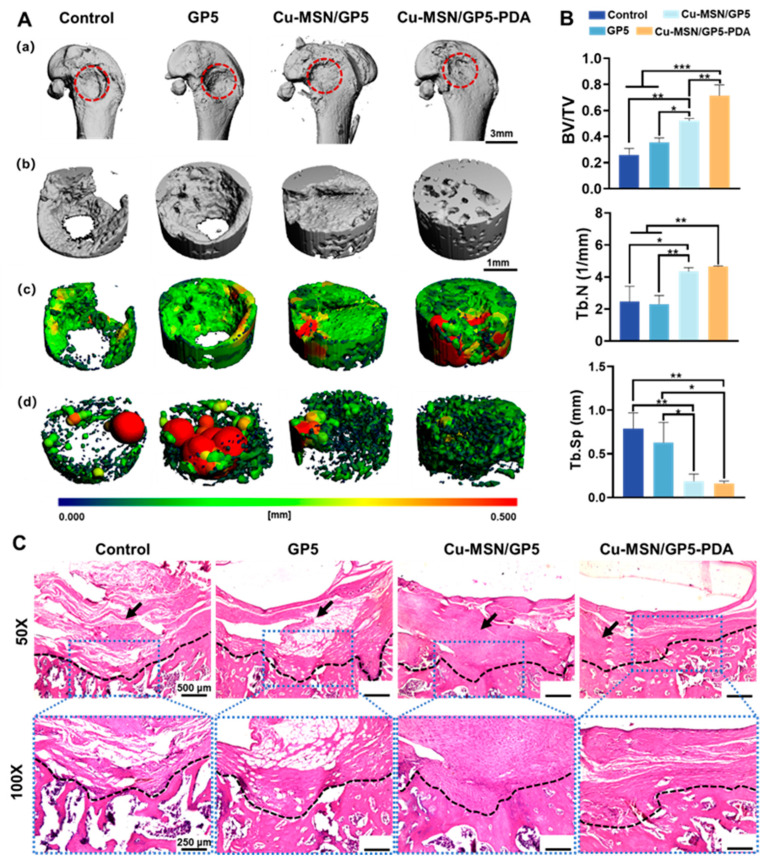
In vivo bone regeneration at 4 weeks after implantation of various membranes in rat femoral condyle defects. (**A**) Micro-CT 3D images of femoral condyles (**a**), new bones (**b**), trabecular thickness (**c**) and trabecular thickness separation (**d**). (**B**) Bone volume/Total volume (BV/TV), trabecular number (Tb. N), and trabecular separation (Tb. Sp). (**C**) Representative histologic analysis of the femoral condylar defect (the black arrows refer to the fiber wrap and the black dotted line indicates the dividing line between bone and fiber tissue). * *p* < 0.05, ** *p* < 0.01, *** *p* < 0.001.

The formation of new bone around the membrane implants was assessed 4 weeks after surgery. Micro-CT imaging revealed that the implantation of Cu–MSN/GP5–PDA membranes led to the most favorable outcome in healing bone defects (Figure 9A(a,b)), showed highest trabecular thickness distribution (Figure 9A(c)) and smallest trabecular separation (Figure 9A(d)). Conversely, the Control and GP5 groups exhibited limited healing with minimal bone tissue growth extending into the center of the defect. Quantitative micro-CT analysis showed that the Cu–MSN/GP5–PDA group achieved the highest levels of total bone tissue formation (BV/TV), trabecular number (Tb.N), and the lowest trabecular separation (Tb.Sp), with the Cu–MSN/GP5 group coming in second (Figure 9B). When the femoral condyle defects were covered with GBR membranes, fibrous connective tissue was prevented from entering the bone defect area and taking up space for bone repair. Therefore, the bone defect repair effect of the three groups covered by GBR membrane was better than that of the control group.

As illustrated in Figure 9C, histological examination was performed to observe the bone regeneration guided by the GBR membranes. No significant inflammation or infection was observed in any of the groups. In both the control and GP5 groups, the femoral condyle defect areas were predominantly occupied by fibrous connective tissue. We can clearly see the boundary between the fibrous tissue and the bone tissue. But in the Cu–MSN/GP5 and Cu–MSN/GP5–PDA groups, there are some newly bone matrix around the bone defect and the latter was more apparent. The results of H&E staining were consistent with the conclusions drawn from micro-CT analysis. Our in vitro findings demonstrate that the copper doped and PDA coated-functionalized membranes enabled the prevention of fibrous connective tissue invasion and bacterial infection.

## 4. Conclusions

In addressing the multifaceted challenges posed by the oral environment, our study underscores the necessity for multifunctional bioactive GBR membranes that cater to elevated bone healing demands. The Cu–MSN/GP–PDA membrane developed in this study presents three groundbreaking advancements over conventional GBR membranes. First, its unique mechanical-degradation equilibrium achieves 11.23 ± 0.91 MPa tensile strength with <30% strength loss over 5 weeks—surpassing collagen membranes (Bio-Gide^®^: 3.7 MPa initial strength, 78% loss at 4 weeks)—while avoiding excessive strength-induced tissue dehiscence (PLGA membranes, metal alloy membranes). Second, the sustained Cu^2+^ release enables dual-phase bioactivity: early-stage antibacterial efficacy without cytotoxicity, followed by prolonged angiogenic-osteogenic stimulation. Compared to single-function commercial membranes requiring additional growth factor coatings or antibiotic supplements, our integrated solution reduces clinical steps while enhancing cost-effectiveness. This research introduces an innovative strategy for oral bone repair and establishes a foundational platform for future advancements in GBR membrane design. Ongoing exploration into the biological impacts and performance enhancement of asymmetric membranes, including the refinement of composition ratios and the incorporation of additional bioactive agents, holds significant promise for further amplifying their efficacy in oral bone regeneration.

## Data Availability

The original contributions presented in the study are included in the article; further inquiries can be directed to the corresponding author.
